# Dogs and Opossums Positive for Vaccinia Virus during Outbreak Affecting Cattle and Humans, São Paulo State, Brazil

**DOI:** 10.3201/eid2202.140747

**Published:** 2016-02

**Authors:** Marina G. Peres, Claudenice B. Barros, Camila M. Appolinário, João M.A.P. Antunes, Mateus S.R. Mioni, Thais S. Bacchiega, Susan D. Allendorf, Acácia F. Vicente, Clóvis R. Fonseca, Jane Megid

**Affiliations:** Universidade Estadual Paulista Júlio de Mesquita Filho, Botucatu, Brazil

**Keywords:** vaccinia virus, viruses, outbreak, hosts, reservoirs, dogs, opossums, zoonoses, PCR, São Paulo State, Brazil

## Abstract

During a vaccinia virus (VACV) outbreak in São Paulo State, Brazil, blood samples were collected from cows, humans, other domestic animals, and wild mammals. Samples from 3 dogs and 3 opossums were positive for VACV by PCR. Results of gene sequencing yielded major questions regarding other mammalian species acting as reservoirs of VACV.

Since the first vaccinia virus (VACV) outbreak in Brazil in 1999, researchers have speculated on the origins and possible reservoirs of VACV (*1*,*2*). Wild and peridomestic rodents are known to be reservoirs of cowpox in Europe (*3*), but in Brazil, their involvement as VACV reservoirs is unclear. Although studies have reported experimental transmission of VACV between rodents and cows (*4*), this finding was not confirmed during outbreaks in Brazil.

The isolation and characterization of a VACV isolate in a peridomestic rodent has been described on a farm in Minas Gerais State, which raised questions about the role of rodents in VACV maintenance in Brazil (*5*). However, a recent serologic study on VACV reservoirs suggested that wild rodents might have a secondary role in VACV maintenance in São Paulo State (*6*).

Despite the absence of reports of clinical signs in dogs and other domestic or wild mammals during VACV outbreaks, in this report, we describe 3 dogs (*Canis lupus familiaris*) and 3 opossums (*Didelphis albiventris*) without clinical signs that were obtained during from a VACV outbreak. Blood samples from these animals were positive by PCR for VACV.

## The Study

This study was approved by the Ethical Committee of Animals Uses in Veterinary Medicine and Animal Production of São Paulo State University. The capture of wild animals was authorized by the Brazilian Institute of Environment and Natural Resources Renewable.

In October 2012, the veterinary service of Itatinga County (23°6′7″S, 48°36′57″W) in São Paulo State, Brazil, reported an outbreak similar to that of bovine smallpox that affected 2 small dairy farms. The Veterinary Medical and Animal Husbandry School of São Paulo State University were contacted, and a team of veterinarians visited the 2 dairy farms to collect samples for diagnosis and relevant information by using an epidemiologic questionnaire.

Both farms (1.5 km apart) used manual milking systems and had similar sanitary management of herds. The animals were vaccinated against foot and mouth disease, brucellosis, and clostridiosis. Ivermectin or albamectin were used for control of endoparasites. Animals were raised in pastures, and there were no changes in management or introduction of new animals on both farms. There were no previous outbreaks on these farms.

**Figure 1:**
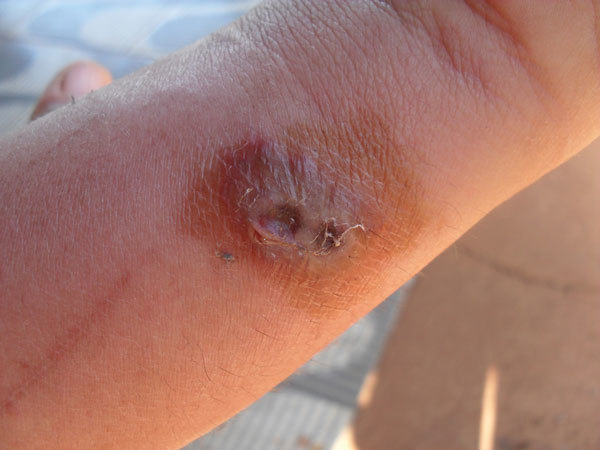
Lesion on arm of a human who worked as a milker, São Paulo State, Brazil. The lesion was determined to be caused by infection with vaccinia virus.

Blood and scab samples from udders of cows or nostrils of calves were collected from 31 affected animals. Serum and whole blood were collected from 2 humans who worked as milkers and had lesions on their hands and arms (Figure 1). Blood samples were also collected from 6 dogs, 6 pigs, 2 horses, 2 rams, 3 opossums, 1 coati (*Nasua nasua*), and 2 wild rodents (*Akodon montensis* and *Nectomys squamipes*). Both domestic and wild mammals were evaluated at the time of collection for characteristic clinical signs of VACV, such as vesicles, scabs, or crusts.

Opossums and the coati were captured by using a trap (Tomahawk Live Trap, Hazelhurst, WI, USA) containing thigh or drumstick chicken as bait. These animals were anesthetized with tiletamine and zolazepam by using the recommended dose for each species (*7*). Wild rodents were captured by using a trap (H.B. Sherman Traps, Inc., Tallahassee, FL, USA) containing peanuts, cornmeal, oats, and canned sardines as bait. These animals were anesthetized in an autoclavable bag containing gauze soaked in ethyl ether and then euthanized by using deep anesthesia. Five trap nights were required to capture animals in the native forest areas surrounding both farms.

Virus DNA was extracted from scabs and total blood samples by using the Invisorb Spin Tissue Mini Kit and the Invisorb Spin Blood Mini Kit (Stratec Molecular, Berlin, Germany), respectively. A seminested PCR was conducted for amplification of the *A56R* gene of VACV (*8*), and positive samples were submitted for gene sequencing at the Federal University of Minas Gerais State (Belo Horizonte, Brazil).

**Figure 2:**
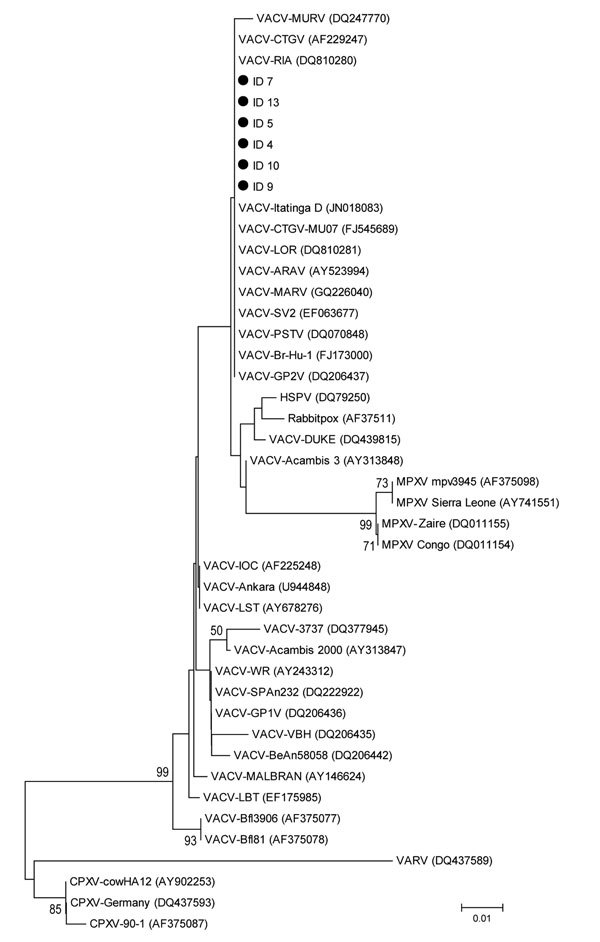
Phylogenetic tree of A56R genes of orthopoxviruses, São Paulo State, Brazil. Tree was constructed by using the neighbor-joining method, the Tamura-3 model of nucleotide substitutions, and 1,000 bootstrap replicates in MEGA 4.0 (http://www.megasoftware.net/mega4/mega.html). Black circles indicate group 1 vaccinia virus (VACV) isolates from this study. Numbers along branches are bootstrap values. ID 5 (KJ741390.1), ID 7 (KJ741391.1), and ID 13 (KJ741392.1) are from opossum (*Didelphis albiventris*) blood samples; ID 5 and ID 7 are from the first farm sampled; ID 13 is from the second farm sampled; ID 4 (KJ741387.1), ID 9 (KJ741388.1), and ID 10 (KJ741389.1) are from canine blood samples; and ID 4, ID 9, and ID 10 are from the first farm sampled. MURV, Muriaé virus; CTGV, Cantagalo virus; ARAV, Araçatuba bat virus; MARV, Mariana virus; PSTV, Passatempo virus; GP2V, Guarani P2 vaccinia virus; HSPV, horsepox virus; MPXV, monkeypox virus; VARV, variola virus; CPXV, cowpox virus. Scale bar indicates nucleotide substitutions per site.

Responses to the epidemiologic questionnaire showed that no factors could be correlated with the 2 outbreaks. No domestic or wild mammals had clinical signs at the time of blood sample collection. However, 3 dogs and 3 opossums were positive by PCR for VACV. Gene sequences obtained (GenBank accession nos. KJ741387.1, KJ741388.1, KJ741389.1, KJ741390.1, KJ741391.1, and KJ741392.1) showed that VACV isolated in this study had the same deletion of six amino acids at position 251 that is found in group I VACVs from Brazil, such as Cantagalo virus, Araçatuba virus, Passatempo virus, and Guarani P2 virus (*4*,*9*,*10*) (Figure 2).

## Conclusions

The fact that the dogs and opossums positive by PCR for VACV did not have clinical signs of infection might indicate only a subclinical infection or may be related to the dichotomy of VACVs from Brazil, which is not only genetic but also biologic. Ferreira et al. (*4*) showed that although there was no difference in severity of lesions in humans and cows affected by group I and II VACVs, BALB/c mice that were intranasally inoculated with group I VACVs did not have clinical signs. In contrast, mice inoculated with group II VACS, such as Belo Horizonte virus and Gurani P1 virus, had acute respiratory illness, followed by death.

More than 1 mammalian species, either wild or domestic, might be acting as a reservoir of group I VACVs from Brazil, possibly acquiring and transmitting the virus without showing clinical signs. This assumption corroborates the findings of Peres et al. (*6*), which showed a high seroprevalence in dogs without clinical signs, questioning their possible role as a reservoir and suggesting more studies to confirm these findings.

Genetic and epidemiologic analysis showed that VACV circulating in this region are members of group I VACVs in Brazil. These findings might support the absence of clinical signs and raise major questions about the potential for >1 mammalian species, either wild or domestic, to act as a reservoir. More studies are needed to further elucidate this ecologic situation.

## References

[1] Damaso CR, Esposito JJ, Condit RC, Moussatche N. An emergent poxvirus from humans and cattle in Rio de Janeiro State: Cantagalo virus may derive from Brazilian smallpox vaccine. Virology. 2000;277:439–49. 10.1006/viro.2000.0603 11080491

[2] Trindade GS, Emerson GL, Carroli DS, Kroon EG, Damon IK. Brazilian vaccinia viruses and their origins. Emerg Infect Dis. 2007;13:965–72 . 10.3201/eid1307.061404 18214166 PMC2878226

[3] Flores EF. Veterinary virology [in Portuguese]. Editora da Universidade Federal de Santa Maria. 2007;18:492–511.

[4] Ferreira JM, Abrahão JS, Drumond BP, Oliveira FM, Alves PA, Pascoal-Xavier MA. Vaccinia virus: shedding and horizontal transmission in a murine model. J Gen Virol. 2008;89:2986–91. 10.1099/vir.0.2008/003947-0 19008383

[5] Abrahão JS, Guedes MI, Trindade GS, Fonseca FG, Campos RK, Mota BF, One more piece in the VACV ecological puzzle: could peridomestic rodents be the link between wildlife and bovine vaccinia outbreaks in Brazi? PLoS ONE. 2009;4:e7428. 10.1371/journal.pone.0007428 19838293 PMC2758550

[6] Peres MG, Bacchiega TS, Appolinario CM, Vicente AF, Allendorf SD, Antunes JM, Serological study of vaccinia virus reservoir in areas with and without official reports of outbreaks in cattle and humans in São Paulo, Brazil. Arch Virol. 2013;158:2433–41. 10.1007/s00705-013-1740-5 23760628 PMC3830743

[7] Nunes AL, Cruz ML, Cortopasso SR. Anesthesiology. In: Cubas SZ, Silva ICR, Catão JL, editors. Treaty for wild animals [in Portuguese]. São Paulo (Brazil): Editoria Roca; 2006. p. 1040–67.

[8] Ropp SL, Jin Q, Knight JC, Massung RF, Esposito JJ. PCR strategy for identification and differentiation of smallpox and other orthopoxviruses. J Clin Microbiol. 1995;33:2069–76 . 7559950 10.1128/jcm.33.8.2069-2076.1995 PMC228337

[9] Trindade GS, Lobato ZI, Drumond BP, Leite JA, Trigueiro RC, Guedes MI, Isolation of two vaccinia virus strains from a single bovine vaccinia outbreak in a rural area from Brazil: implications on the emergence of zoonotic orthopoxviruses. Am J Trop Med Hyg. 2006;75:486–90 . 16968926

[10] Drumond BP, Leite JA, Fonseca FG, Bonjardim CA, Ferreira PC, Kroon EG. Brazilian virus strains are genetically divergent and differ from the Lister vaccine strain. Microbes Infect. 2008;10:185–97. 10.1016/j.micinf.2007.11.005 18248758

